# Supplementary material for: Multiple time measurements of multidimensional psychiatric states from immediately before the COVID-19 pandemic to one year later: a longitudinal online survey of the Japanese population
Source: Transl Psychiatry. 2021 Nov 11;11:573. doi: 10.1038/s41398-021-01696-x (PMC8581018; doi:10.1038/s41398-021-01696-x)
Supplement: Supplementary file 1 — Supplementary material [file 41398_2021_1696_MOESM1_ESM.pdf]

Supplementary Methods

Details of participant recruitment

This study was originally planned in 2019 and later extended to examine the psychiatric impact of COVID-19. The purpose of the original study in 2019 was to investigate the relationship between smartphone addiction and multiple psychiatric states. We relied on Macromill, INC. (https://monitor.macromill.com/), the largest online research company in Japan for participant recruitment. This company maintains a participant pool of about 1.2 million individuals in Japan. Among this participant pool, 99,156 randomly chosen individuals, aged 18 and above, living in the Kansai region of Japan, were invited via e-mail to participate in screening for the original study before the COVID-19 pandemic. Our invitation e-mail for the survey contained information about informed consent. The completion of the entire questionnaire was considered to indicate participant consent. 5,955 respondents passed the screening and were recruited, such that the population included equal ratios of individuals belonging to each quintile in the problematic smartphone survey. Participants received points that could be redeemed as money after completing the survey. In response to the COVID-19 pandemic, individuals from this survey were invited to participate in follow-up surveys that took place in August 2020 (T1), December 2020 (T2), and April 2021 (T3). People who responded to only the baseline survey numbered 1,647.

The strategy for determining the timing of acquisition was to acquire data near the peaks of the second and third waves when the spread of infection became particularly pronounced. Because this interval between T1 and T2 was four months, we decided to acquire data every four months thereafter to keep a constant sampling rate. Coincidentally, the data acquisition period of T2 and T3 by overlapped with the peaks of the fourth and fifth waves of the pandemic. Therefore, we could compare psychiatric states around the peaks of each wave (first to fourth) of the pandemic while keeping the sampling rate constant.

Participants exclusion criteria

We excluded 326 individuals because of inconsistencies or contradictions in their answers, such as, sleep time was the same as wake-up time, gender inconsistencies from survey to survey, contradictions in regard to drinking in the Alcohol Use Disorders Identification Test (AUDIT) (e.g., A participant answered "Never" in 1st question "How often do you have a drink containing alcohol?", but answered "3 or 4" or more in the second question, "How many drinks containing alcohol do you have on a typical day when you are drinking?" An additional 419 individuals were excluded because they responded identically to all items, using only the maximum or minimum values in the questionnaires, including reversed questions (i.e., in CES-D, STAI-Y, AQ).

Details of the questionnaires

Major depression disorder was measured using the Center for Epidemiologic Studies Depression Scale (CES-D), internal consistency of 0.85 in the original version[1]. Construct validity has been confirmed with Hamilton Clinician's Rating scale (r = 0.44) and the Raskin Rating scale(r = 0.54)[1]. Its reliability and validity have been confirmed in Japanese (Cronbach's α = 0.80)[2]. General anxiety was measured with the State-Trait anxiety scale (STAI)[3], which has a Cronbach's alpha of 0.86 to 0.95 in the original study. Reliability and validity have been confirmed in Japanese (Cronbach's α = 0.92)[4]. Social anxiety was measured using the Liebowitz Social Anxiety Scale (LSAS-fear/avoid)[5], a Cronbach's alpha of 0.95 in the original version. Construct validity has been confirmed with Safren's four-factor model in the original version[5] and Social Avoidance and Distress Scale and Professional Diagnosis in the Japanese version[6]. Obsessive-compulsive disorder was measured using the Obsessive-Compulsive Inventory (OCI), a Cronbach's alpha of 0.86 to 0.95 in the original version[7] and 0.96 in the Japanese version[8]. Construct validity has been confirmed with Yale-Brown Obsessive Compulsive Scale, Compulsive Activity Checklist, and Maudslay Obsessive-Compulsive Inventory in the original version and Maudsley Obsessive-Compulsive Inventory (r = 0.74), STAI (r = 0.41), and CES-D (r = 0.48) in the Japanese version[8]. Alcohol dependence was measured using the Alcohol Use Disorders Identification Test (AUDIT), a Cronbach's alpha of 0.65-0.93 in the original version[9]. Its reliability and validity have been confirmed in Japanese (Cronbach's α = 0.81, construct validity was confirmed using a structured interview[10]. Internet-related problems were measured using the Compulsive Internet Use Scale (CIUS), a Cronbach's alpha of 0.89[11] in the original version and more than 0.9 in the Japanese version[12]. Construct validity has been confirmed by the strong positive correlation with the Online Cognition Scale ($r = 0.70$, $p < 0.001$) and the amount of time spent online (r = 0.33, $p < 0.001$) in the original version and POSI, MR, DSR, NO, K6 and UCLA Loneliness in the Japanese version. Autism Spectrum Disorder (ASD) was measured using the Autism-Spectrum Quotient (AQ), a Cronbach's alpha of 0.63~0.77 in the original version[13] and internal consistency of 0.81 in the

Japanese version[14]. Construct validity has been confirmed with professional diagnosis based on DSM-IV (r = 0.58) and the experience of exposure to social adaptation problems (r = 0.92) in the Japanese version[14]. Adult attention-deficit/hyperactivity disorder (ADHD) was measured using the Adult ADHD Self-Report Scale (ASRS)[15], a Cronbach's alpha of 0.88 to 0.89 in the original version[16], 0.83 in the Japanese version[17]. Construct validity has been confirmed by the Japanese version of the Conners' Adult ADHD Rating Scales-Self Report and Beck Depression Inventory-II in the Japanese version[17]. These psychiatric disorders affect Internet-related problems[18–23].

For the original study, we assessed other scales and excluded them from the analysis to reduce redundancies in the variables. STAI-Y-trait, Internet gaming disorder (IGDS), and Internet addiction test (IAT) were also assessed for the original study purpose. In this study, these scores were excluded from the analysis to reduce redundancies in the variables: STAI-Y-trait was excluded because we measured the STAI-Y-state. IGDS-J and IAT were excluded because we measured the CIUS.

Model specification of generalized linear model regression

model1: ΔPC1(T1-T0) = Intercept + PC1(T0) + PC2 (T0) + PC3 (T0) + PC4 (T0) + sex + age + marital status + age of youngest child in household + number of cohabitants + household income + employment status + the status of COVID-19 infection + changes in the amount of face-to-face communication with family from T0 to T1 + changes in the amount of online communication with family from T0 to T1 + number of received government compensation payments + impact of income because of the pandemic

model2: ΔPC2(T3-T0) = Intercept + PC1(T0) + PC2 (T0) + PC3 (T0) + PC4 (T0) + sex + age + marital status + age of youngest child in household + number of cohabitants + household income + employment status + the status of COVID-19 infection + changes in the amount of face-to-face communication with family from T0 to T3 + changes in the amount of online communication with family from T0- to T3 + number of received government compensation payments + impact of income because of the pandemic

model3: ΔPC4(T1-T0) = Intercept + PC1(T0) + PC2 (T0) + PC3 (T0) + PC4 (T0) + sex + age + marital status + age of youngest child in household + number of cohabitants + household income + employment status + the status of COVID-19 infection + changes in the amount of face-to-face communication with family from T0 to T1 + changes in the amount of online communication with family from T0 to T1 + number of received government compensation payments + impact of income because of the pandemic

Supplementary analysis

This analysis was recalculated from data for each condition in sex and age to clarify the robusticity of the data structure for inter-conditions. The results are shown in Supplementary Figure 5 ~ 7.

Supplementary Table 1 A list of COVID-19 pandemic related questions

Condition	Instruction		Items
**Communication with family by face-to-face or online**	We would like to ask you about communication between you and your family.<br>(1) How much time do you spend communicating face-to-face in a day ?<br>(2)How much time do you spend communicating online (e.g. on LINE) in a day ?	1	No communication
		2	less than 15 minutes
		3	15 minutes to less than 30 minutes
		4	30 minutes to less than 1 hour
		5	more than 1 hour
**COVID-19 infection**	Which of the following best describes your situation to date regarding COVID-19 infection?	1	I was diagnosed as with covid-19 infection, and my test results were positive.
		2	I was diagnosed with covid-19 infection, but I did not get tested.
		3	I have not been diagnosed with covid-19 infection, but I am convinced that I was positive.
		4	I have not been diagnosed with covid-19 infection, but I think I was probably positive.
		5	I have not been diagnosed with covid-19 infection, but I think I was probably negative.
		6	I have not been diagnosed with covid-19 infection, and I am convinced that I was negative.
**COVID-19 economic impact†**	What kind of economic impact has the new coronavirus epidemic had on you ?<br><br>Please set the number since the last survey to 0, and select a negative percentage if the impact has decreased, or a positive percentage if it has increased.<br>(Required except for others)	1	More than 100% reduction
		2	81%～100% reduction
		3	61%～80% reduction
		4	41%～60% reduction
		5	21%～40% reduction
		6	1%～20% reduction
		7	No change
		8	1%～20% increase
		9	21%～40% increase
		10	41%～60% increase
		11	61%～80% increase
		12	81%～100% increase
		13	Increase by more than 100%
**COVID-19 received insurance programs††**	Please tell us about any insurance programs you have received since the last survey. (Choose as many as you like)	1	The Special Cash Payment (related to emergency economic treatment for Covid-19) [Individual]
		2	Support for parents responding to temporary closures of elementary schools and other schools [Individual]
		3	Support for users of company-led baby-sitters (special treatment) [Individuals and corporations]
		4	Financial support for reduction and exemption of national health insurance premiums (taxes) [Individuals and Sole proprietors]
		5	Cash payment for sustainability [Individuals and Corporations]
		6	Subsidy for employment adjustment (special treatment for Covid-19) [Individuals and Corporations]
		7	Subsidy for responding to the closure of elementary schools for Covid-19 [Corporations]
		8	Subsidy for promoting productivity improvement in manufacturing, commerce, and services (special case) 【Corporations】
		9	Subsidy for sustaining small-scale business 【Sole proprietors and Corporations】
		10	IT introduction subsidy (Category C, special case) 【Corporations】
		11	Support from your company (e.g., you were paid to stay at home)
		12	Other support from the government
		13	Other support from the prefecture
		14	Other support from municipalities
		15	Other
		16	None in particular

† When analysing, the values were inverted to interpret easily

†† We summed up the number of these choices to create the index of the number of compensations.

Supplementary Table 2 Characteristics of the survey population (N = 3935)

**All**	3935 (100%)							
**Gender**	Male	Female						
	2034 (52%)	1901 (48%)						
**Marital status**	Not married	Married						
	1386 (35%)	2549 (65%)						
**Age of youngest child in household**	No children	0~3	4~6	7~9	10~12	13-15	16-18	19~
	2134 (54%)	339 (9%)	160 (4%)	174 (4%)	184 (5%)	183 (5%)	160 (4%)	601 (15%)
**Household income**	Lowest	2nd	3rd	4th	Highest	Missing		
	961 (24%)	858 (22%)	687 (17%)	353 (9%)	672 (17%)	404 (10%)		
**Job**	No job	Self-employed	Employee	Other				
	649 (16%)	882 (22%)	2069 (53%)	335 (9%)				

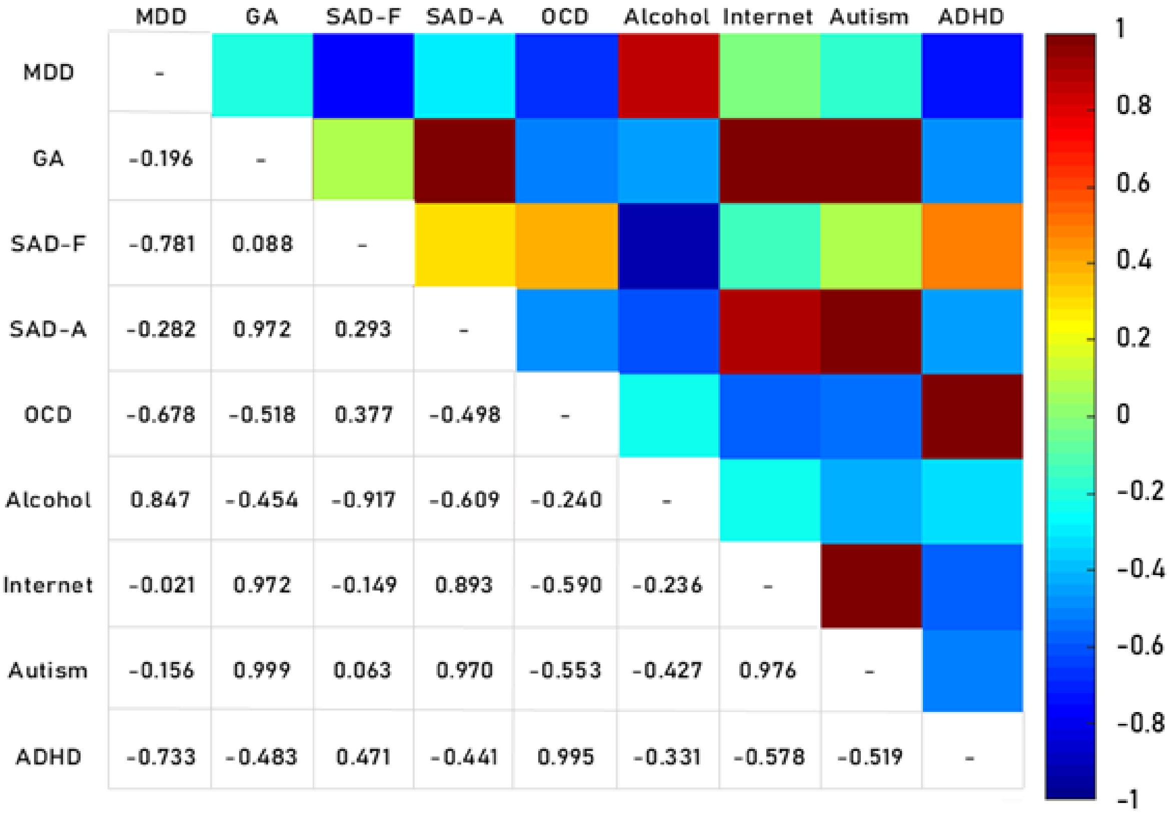

Supplementary Figure 1 Correlation matrices for changes in scores of psychiatric disorders over time MDD: major depressive disorder as measured with the CES-D; GA: general anxiety as measured with the STAI-Y; SAD-F: fear aspects of social anxiety disorder as measured using the LSAS-F; SAD-A: avoidance aspects of social anxiety disorder as measured with the LSAS-A; OCD: obsessive-compulsive disorder as measured with the OCI; Alcohol: alcohol-related problems as measured using the AUDIT; Internet: internet-related problems as measured with the CIUS; ASD: autism as measured using the AQ; ADHD: attention deficit and hyperactivity disorders as measured using the ASRS.

Supplementary Table 3 *p* values of each psychiatric exacerbation

	MDD	GA	SAD-F	SAD-A	OCD	Alcohol	Internet	ASD	ADHD
T0-T1	>0.8	<0.001	>0.8	<0.001	>0.8	>0.8	<0.001	>0.8	<0.001
T0-T2	<0.001	<0.001	>0.8	<0.001	>0.8	>0.8	<0.001	>0.8	>0.8
T0-T3	<0.001	<0.001	>0.8	<0.001	0.180	0.192	<0.001	>0.8	0.025
T1-T2	<0.001	0.539	>0.8	0.122	>0.8	0.431	0.059	>0.8	0.029
T1-T3	<0.001	>0.8	0.799	>0.8	>0.8	0.037	<0.001	>0.8	>0.8
T2-T3	>0.8	>0.8	>0.8	0.003	>0.8	>0.8	>0.8	>0.8	>0.8

MDD: major depressive disorder as measured with the CES-D; GA: general anxiety as measured using the STAI-Y; SAD-F: fear aspects of social anxiety disorder as measured with the LSAS-F; SAD-A: avoidance aspects of social anxiety disorder as measured with the LSAS-A; OCD: obsessive-compulsive disorder as measured using the OCI; Alcohol: alcohol-related problems as measured with AUDIT; Internet: internet-related problems as measured using the CIUS; ASD: autism as measured with the AQ; ADHD: attention deficit and hyperactivity disorders as measured using the ASRS. Tx-Tx represents the difference between two-time points. *P* values were adjusted by Bonferroni correction.

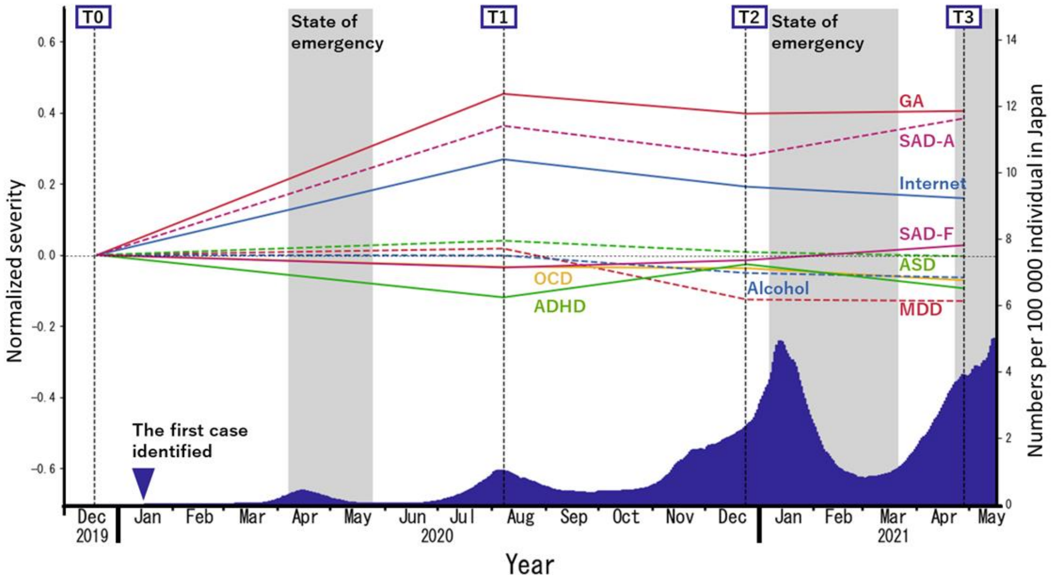

Supplementary Figure 2 Trajectories of each psychiatric score in the survey population (N = 3935)
The blue area under the curve represents a 15-day moving average of daily new cases of COVID-19 per 100 000 Japanese residents.
GA: general anxiety as measured with the STAI-Y; SAD-A: avoidance aspects of social anxiety disorder as measured using the LSAS-A; Internet: internet-related problems as measured using the CIUS; ASD: autism as measured with the AQ; SAD-F: fear aspects of social anxiety disorder as measured using the LSAS-F; OCD: obsessive-compulsive disorder as measured with the OCI; ADHD: attention deficit and hyperactivity disorders as measured with the ASRS; Alcohol: alcohol-related problems as measured using the AUDIT; MDD: major depressive disorder as measured with the CES-D.

Supplementary Table 4 Characteristics of the study population and each PC loading in the study population (N = 2274)

	Sample size	%	PC1 mean (SD)	PC2 mean (SD)	PC3 mean (SD)	PC4 mean (SD)
**All**	2274	100%	0.000 (0.021)	0.000 (0.021)	0.000 (0.021)	0.000 (0.021)
**Gender**						
Male	1225	54%	0.066 (0.029)	-0.236 (0.027)	0.131 (0.031)	0.091 (0.028)
Female	1049	46%	-0.077 (0.030)	0.276 (0.031)	-0.153 (0.027)	-0.106 (0.031)
**Mrital status**						
Not married	801	35%	0.154 (0.038)	0.029 (0.036)	-0.211 (0.035)	0.050 (0.037)
Married	1473	65%	-0.084 (0.025)	-0.016 (0.026)	0.115 (0.026)	-0.027 (0.025)
**Age of youngest child in household**						
No children	1254	55%	0.017 (0.029)	0.037 (0.028)	-0.060 (0.029)	-0.025 (0.028)
0~3	160	7%	0.080 (0.071)	0.105 (0.078)	-0.120 (0.076)	-0.132 (0.081)
4~6	90	4%	0.115 (0.105)	-0.186 (0.113)	-0.029 (1.009)	-0.111 (0.096)
7~9	101	4%	0.038 (0.100)	-0.037 (0.101)	0.043 (1.099)	0.121 (0.109)
10~12	106	5%	-0.074 (0.085)	-0.072 (0.095)	0.089 (0.003)	0.086 (0.098)
13-15	100	4%	0.132 (0.096)	-0.224 (0.094)	0.111 (0.011)	0.072 (0.099)
16-18	101	4%	-0.073 (0.104)	-0.219 (0.094)	0.216 (0.000)	0.191 (0.103)
19~	362	16%	-0.128 (0.048)	0.026 (0.052)	0.026 (0.052)	0.040 (0.051)
**Household income**						
Lowest	567	25%	0.159 (0.043)	0.093 (0.045)	-0.071 (0.042)	-0.040 (0.045)
2nd	495	22%	-0.039 (0.044)	0.020 (0.040)	-0.067 (0.041)	-0.018 (0.043)
3rd	409	18%	-0.016 (0.048)	-0.108 (0.048)	0.048 (0.050)	0.049 (0.047)
4th	222	10%	-0.167 (0.063)	-0.257 (0.065)	0.279 (0.070)	0.063 (0.063)
Highest	382	17%	-0.094 (0.052)	0.028 (0.051)	0.028 (0.051)	0.031 (0.051)
Missing	199	9%	0.042 (0.066)	0.140 (0.073)	-0.214 (0.067)	-0.075 (0.075)
**Job**						
No job	375	16%	0.078 (0.055)	0.204 (0.052)	-0.156 (0.048)	-0.068 (0.052)
Self-employed	517	23%	0.026 (0.029)	-0.182 (0.028)	0.083 (0.030)	0.069 (0.029)
Employee	1177	52%	-0.153 (0.042)	0.205 (0.043)	-0.021 (0.043)	-0.095 (0.044)
Other	205	9%	0.095 (0.070)	0.154 (0.068)	-0.135 (0.068)	-0.034 (0.075)

PC1: Principal component 1; PC2: Principal component 2; PC3: Principal component 3; PC4: Principal component 4.

Supplementary Table 5 Characteristics of the study population and each PC loading in the survey population (N = 3935)

	Sample size	%	PC1 mean (SD)	PC2 mean (SD)	PC3 mean (SD)	PC4 mean (SD)
**All**	3935	100%	0.000 (0.016)	0.000 (0.016)	0.000 (0.016)	0.000 (0.016)
**Gender**						
Male	2034	52%	0.087 (0.022)	-0.261 (0.021)	0.146 (0.024)	0.082 (0.022)
Female	1901	48%	-0.093 (0.022)	0.279 (0.023)	-0.156 (0.020)	-0.088 (0.023)
**Mrital status**						
Not married	1386	35%	0.172 (0.029)	0.011 (0.027)	-0.154 (0.027)	0.048 (0.028)
Married	2549	65%	-0.094 (0.019)	-0.006 (0.020)	0.084 (0.019)	-0.026 (0.019)
**Age of youngest child in household**						
No children	2134	54%	0.055 (0.023)	0.025 (0.022)	-0.055 (0.022)	-0.027 (0.022)
0~3	339	9%	-0.008 (0.050)	0.022 (0.052)	-0.121 (0.051)	-0.116 (0.056)
4~6	160	4%	-0.033 (0.076)	-0.156 (0.078)	-0.007 (0.079)	-0.120 (0.070)
7~9	174	4%	0.023 (0.074)	-0.097 (0.074)	0.084 (0.075)	0.109 (0.075)
10~12	184	5%	-0.077 (0.066)	-0.044 (0.071)	0.128 (0.076)	0.092 (0.073)
13-15	183	5%	0.020 (0.067)	-0.115 (0.071)	0.012 (0.076)	0.089 (0.071)
16-18	160	4%	-0.089 (0.078)	-0.129 (0.077)	0.184 (0.087)	0.213 (0.078)
19~	601	15%	-0.146 (0.038)	0.054 (0.042)	0.149 (0.037)	0.051 (0.039)
**Household income**						
Lowest	961	24%	0.152 (0.033)	0.084 (0.034)	-0.071 (0.032)	-0.034 (0.035)
2nd	858	22%	-0.034 (0.034)	0.027 (0.032)	-0.050 (0.033)	-0.001 (0.033)
3rd	687	17%	0.003 (0.036)	-0.101 (0.037)	0.061 (0.039)	0.041 (0.038)
4th	353	9%	-0.226 (0.051)	-0.231 (0.050)	0.157 (0.053)	0.042 (0.049)
Highest	672	17%	-0.099 (0.039)	-0.013 (0.039)	0.102 (0.039)	-0.014 (0.037)
Missing	404	10%	0.067 (0.048)	0.136 (0.051)	-0.136 (0.048)	0.000 (0.050)
**Job**						
No job	649	16%	0.033 (0.040)	0.241 (0.038)	-0.155 (0.034)	-0.046 (0.041)
Self-employed	882	22%	0.041 (0.022)	-0.197 (0.021)	0.091 (0.023)	0.065 (0.021)
Employee	2069	53%	-0.176 (0.032)	0.220 (0.034)	-0.050 (0.032)	-0.100 (0.033)
Other	335	9%	0.144 (0.058)	0.171 (0.058)	-0.130 (0.054)	-0.053 (0.059)

PC1: Principal component 1; PC2: Principal component 2; PC3: Principal component 3; PC4: Principal component 4.

Supplementary table 6 *p* values of each principal component during the pandemic

	PC1	PC2	PC3	PC4
**T0-T1**	<0.001	<0.001	>0.8	<0.001
**T0-T2**	<0.001	<0.001	>0.8	<0.001
**T0-T3**	<0.001	<0.001	>0.8	<0.001
**T1-T2**	0.775	>0.8	>0.8	<0.001
**T1-T3**	>0.8	0.005	>0.8	0.023
**T2-T3**	>0.8	0.001	>0.8	>0.8

PC1: Principal component 1; PC2: Principal component 2; PC3: Principal component 3; PC4: Principal component 4. *p* values were adjusted by Bonferroni correction.

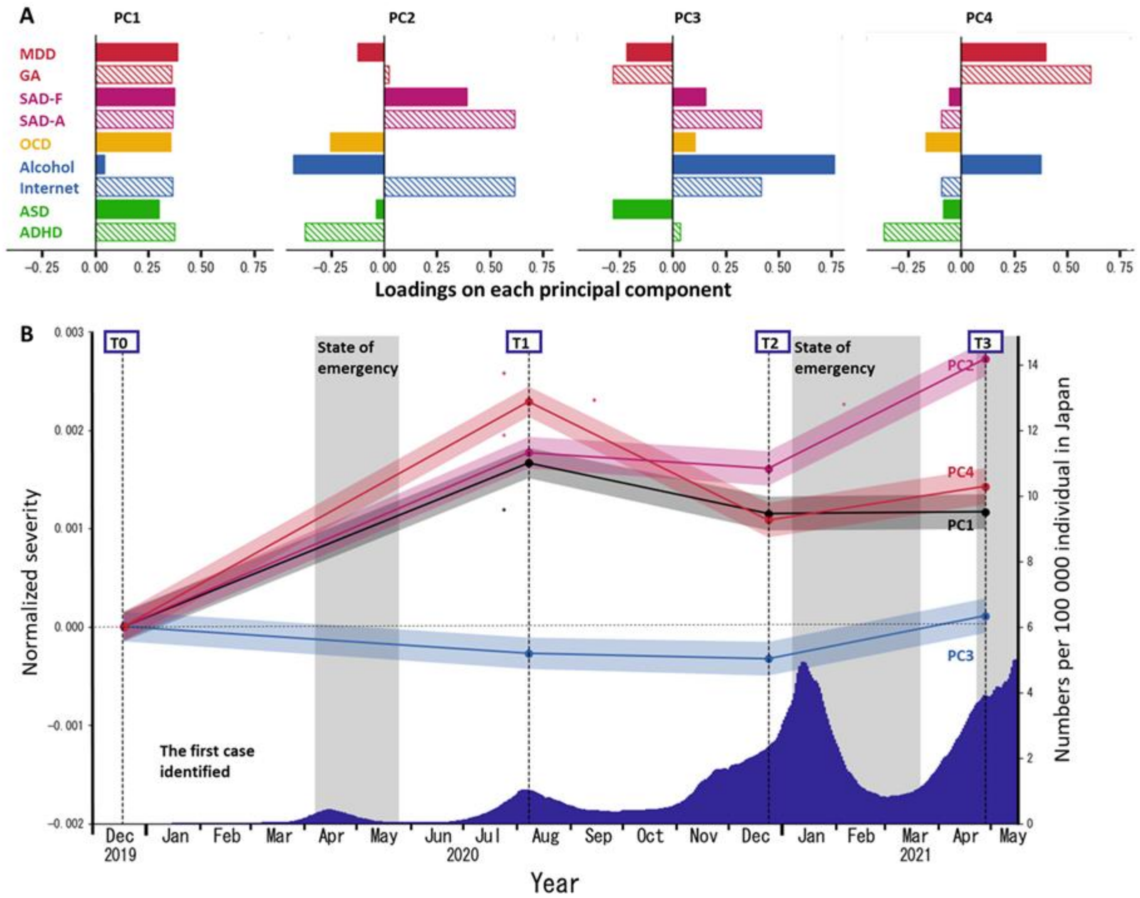

Supplementary Figure 3 Trajectories of PC scores obtained from multidimensional psychiatric scores in the survey population (N = 3935) **A.** Loadings of psychiatric disorder scores for each principal component. **B.** Trajectories of the average of each PC are shown. The blue area under the curve represents a 15-day moving average of daily new COVID-19 cases per 100 000 Japanese residents. Signs of PCs were arranged so that each maximum loading assumed a positive value. Asterisks indicate significant changes in PC score from the previous time point (p<.05, Bonferroni-corrected). All PC scores during the pandemic (T1, T2, and T3) except those of PC3 are significantly higher than the scores pre-pandemic (T0).

MDD: major depressive disorder as measured with the CES-D; GA: general anxiety as measured using the STAI-Y state, SAD-F: fear aspects of social anxiety disorder as measured using the LSAS-F; SAD-A: avoidance aspects of social anxiety disorder as measured using the LSAS-A; OCD: obsessive-compulsive disorder as measured with the OCI; Alcohol: alcohol-related problems as measured using the AUDIT; Internet: internet-related problems as measured with the CIUS; ASD: autism as measured with the AQ; ADHD: attention deficit and hyperactivity disorders as measured with the ASRS

Supplementary Table 7. Statistical values of fixed-effects regression analyses showing within-person changes in multi-dimensional psychiatric status during the pandemic (N = 2274).

	PC1 estimate	PC2 estimate	PC4 estimate	PC1 *p* Value	PC2 *p* Value	PC4 *p* Value
**Psychiatric states before pandemic**						
PC1	-0.35 (0.02)	-0.16 (0.02)	-0.16 (0.02)	<0.001	<0.001	<0.001
PC2	-0.09 (0.02)	-0.40 (0.02)	-0.05 (0.02)	<0.001	<0.001	0.017
PC3	-0.07 (0.02)	-0.10 (0.02)	0.14 (0.02)	0.001	<0.001	<0.001
PC4	0.06 (0.02)	-0.03 (0.02)	-0.46 (0.02)	0.010	0.250	<0.001
**Gender**						
Male				(ref)	(ref)	(ref)
Female	0.32 (0.05)	0.38 (0.04)	0.07 (0.04)	< 0.001	< 0.001	0.325
**Age**	0.03 (0.02)	0.07 (0.02)	0.05 (0.02)	>0.8	0.010	0.084
**Number of cohabitants**	0.01 (0.03)	-0.04 (0.03)	0.01 (0.03)	>0.8	0.558	>0.8
**Marital status**						
Not married				(ref)	(ref)	(ref)
Married	-0.03 (0.05)	0.09 (0.05)	-0.09 (0.05)	>0.8	0.206	0.258
**Age of youngest child in household**						
No children				(ref)	(ref)	(ref)
0~3	0.01 (0.09)	-0.16 (0.09)	0.04 (0.09)	>0.8	0.228	>0.8
4~6	-0.06 (0.11)	-0.14 (0.11)	0.06 (0.11)	>0.8	0.625	>0.8
7~9	0.07 (0.11)	-0.01 (0.10)	0.06 (0.10)	>0.8	>0.8	>0.8
10~12	0.11 (0.10)	0.05 (0.10)	0.07 (0.10)	>0.8	>0.8	>0.8
13-15	0.19 (0.10)	0.06 (0.10)	0.04 (0.10)	0.199	>0.8	>0.8
16-18	-0.13 (0.10)	0.01 (0.10)	-0.02 (0.10)	0.664	>0.8	>0.8
19~	-0.09 (0.07)	0.01 (0.06)	0.06 (0.06)	0.577	>0.8	>0.8
**Household income**						
Lowest				(ref)	(ref)	(ref)
2nd	-0.02 (0.06)	-0.01 (0.06)	-0.12 (0.05)	>0.8	>0.8	0.096
3rd	-0.01 (0.06)	0.00 (0.06)	-0.11 (0.06)	>0.8	>0.8	0.205
4th	-0.08 (0.08)	-0.13 (0.08)	-0.16 (0.07)	>0.8	0.267	0.073
Highest	-0.01 (0.06)	-0.05 (0.06)	-0.05 (0.06)	>0.8	>0.8	>0.8
Missing	0.01 (0.08)	0.02 (0.07)	-0.03 (0.07)	>0.8	>0.8	>0.8
**Job**						
No job				(ref)	(ref)	(ref)
Self-employed	-0.08 (0.06)	-0.16 (0.06)	0.01 (0.06)	0.574	0.017	>0.8
Employee	0.13 (0.06)	0.14 (0.06)	0.06 (0.06)	0.117	0.059	>0.8
Other	0.08 (0.08)	-0.04 (0.08)	-0.08 (0.08)	>0.8	>0.8	>0.8
**COVID-19 infection**						
Negative				(ref)	(ref)	(ref)
Positive	0.10 (0.30)	-0.38 (0.27)	-0.02 (0.29)	>0.8	0.463	>0.8
**Economical impact of COVID-19**						
Impact of income	0.08 (0.02)	0.03 (0.02)	0.06 (0.02)	<0.001	0.330	0.007
Number of compensation	-0.02 (0.02)	-0.01 (0.02)	-0.01 (0.02)	0.695	>0.8	>0.8
**Face communication time difference**						
No change				(ref)	(ref)	(ref)
Increase	-0.04 (0.05)	-0.14 (0.05)	0.03 (0.04)	>0.8	0.007	>0.8
Decrease	0.00 (0.05)	-0.12 (0.05)	0.05 (0.05)	>0.8	0.034	>0.8
**Online communication time difference**						
No change				(ref)	(ref)	(ref)
Increase	-0.07 (0.05)	-0.08 (0.05)	-0.01 (0.05)	0.545	0.347	>0.8
Decrease	0.05 (0.05)	-0.14 (0.04)	0.01 (0.04)	>0.8	0.005	>0.8

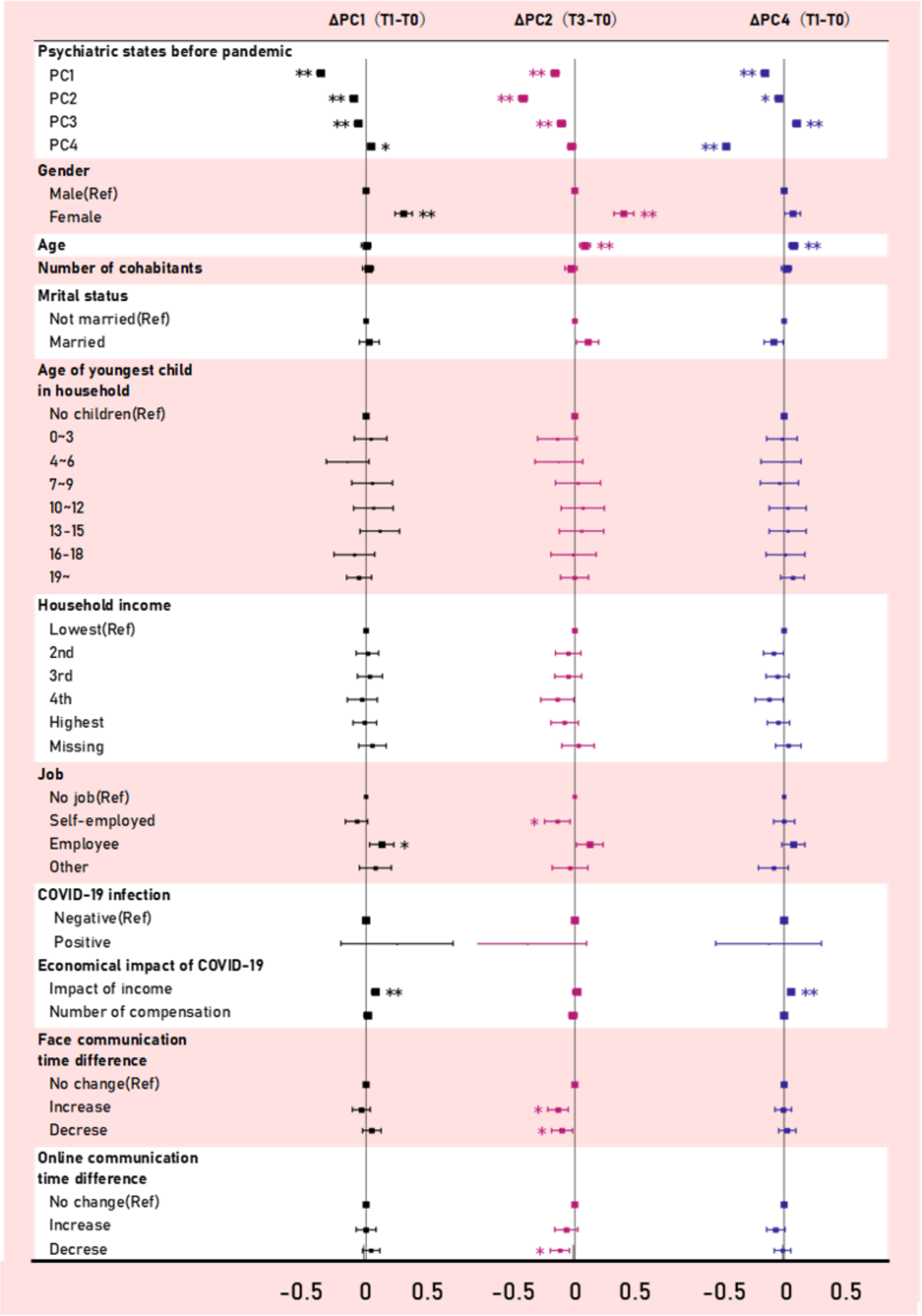

Supplementary Figure 4. Fixed-effects regression analyses showing within-person changes in multi-dimensional psychiatric status during the pandemic (N = 3935)

Forest plots show results of fixed-effects regression analyses. Each plot represents standardized beta coefficients with standard errors. Asterisks indicate statistical significance (* $p < 0.05$, ** $p < 0.001$).Forest plots show results of fixed-effects regression analyses. Each plot represents standardized beta coefficients with standard errors. Asterisks indicate statistical significance (* $p < 0.05$, ** $p < 0.001$)

Supplementary Table 8. Statistical values of fixed-effects regression analyses showing within-person changes in multidimensional psychiatric status during the pandemic (N = 3935).

	PC1 estimate	PC2 estimate	PC4 estimate	PC1 *p* Value	PC2 *p* Value	PC4 *p* Value
**Psychiatric states before pandemic**						
PC1	-0.37 (0.02)	-0.16 (0.02)	-0.16 (0.01)	<0.001	<0.001	<0.001
PC2	-0.10 (0.02)	-0.42 (0.02)	-0.42 (0.01)	<0.001	<0.001	0.017
PC3	-0.06 (0.02)	-0.11 (0.02)	-0.11 (0.01)	0.002	<0.001	<0.001
PC4	0.04 (0.02)	-0.03 (0.02)	-0.03 (0.01)	0.015	0.397	<0.001
**Gender**						
Male				(ref)	(ref)	(ref)
Female	0.31 (0.04)	0.40 (0.04)	0.07 (0.03)	< 0.001	< 0.001	0.086
**Age**	0.00 (0.02)	0.08 (0.02)	0.08 (0.02)	0.421	<0.001	<0.001
**Number of cohabitants**	0.02 (0.02)	-0.03 (0.02)	0.02 (0.02)	>0.8	0.675	0.796
**Mrital status**						
Not married				(ref)	(ref)	(ref)
Married	0.03 (0.04)	0.11 (0.05)	-0.08 (0.04)	>0.8	0.078	0.106
**Age of youngest child in household**						
No children				(ref)	(ref)	(ref)
0~3	0.04 (0.07)	-0.14 (0.08)	-0.01 (0.06)	>0.8	0.234	>0.8
4~6	-0.15 (0.09)	-0.13 (0.10)	-0.02 (0.08)	0.269	0.568	>0.8
7~9	0.05 (0.08)	0.03 (0.09)	-0.03 (0.08)	>0.8	>0.8	>0.8
10~12	0.06 (0.08)	0.06 (0.09)	0.03 (0.08)	>0.8	>0.8	>0.8
13-15	0.11 (0.08)	0.06 (0.09)	0.03 (0.08)	0.483	>0.8	>0.8
16-18	-0.09 (0.08)	-0.01 (0.09)	0.01 (0.08)	>0.8	>0.8	>0.8
19~	-0.06 (0.05)	0.00 (0.06)	0.07 (0.05)	>0.8	>0.8	0.440
**Household income**						
Lowest				(ref)	(ref)	(ref)
2nd	0.01 (0.05)	-0.05 (0.05)	-0.08 (0.04)	>0.8	>0.8	0.175
3rd	0.03 (0.05)	-0.05 (0.06)	-0.05 (0.05)	>0.8	>0.8	>0.8
4th	-0.03 (0.06)	-0.14 (0.07)	-0.12 (0.06)	>0.8	0.116	0.136
Highest	-0.01 (0.05)	-0.08 (0.06)	-0.04 (0.05)	>0.8	0.428	>0.8
Missing	0.05 (0.06)	0.03 (0.07)	0.04 (0.05)	>0.8	>0.8	>0.8
**Job**						
No job				(ref)	(ref)	(ref)
Self-employed	-0.07 (0.05)	-0.14 (0.05)	0.00 (0.04)	0.329	0.020	>0.8
Employee	0.13 (0.05)	0.12 (0.06)	0.08 (0.05)	0.030	0.086	0.268
Other	0.08 (0.07)	-0.04 (0.07)	-0.08 (0.06)	0.750	>0.8	0.537
**COVID-19 infection**						
Negative				(ref)	(ref)	(ref)
Positive	0.26 (0.23)	-0.39 (0.25)	-0.12 (0.23)	>0.8	0.350	>0.8
**Economical impact of COVID-19**						
Impact of income	0.08 (0.02)	0.02 (0.02)	0.02 (0.01)	<0.001	>0.8	0.001
Number of compensation	0.01 (0.02)	-0.02 (0.02)	-0.02 (0.01)	>0.8	>0.8	0.461
**Face communication time difference**						
No change				(ref)	(ref)	(ref)
Increase	-0.04 (0.04)	-0.14 (0.04)	-0.14 (0.01)	>0.8	0.004	>0.8
Decrese	0.05 (0.04)	-0.10 (0.04)	-0.10 (0.01)	0.617	0.047	>0.8
**Online communication time difference**						
No change				(ref)	(ref)	(ref)
Increase	0.00 (0.04)	-0.07 (0.05)	-0.07 (0.01)	>0.8	0.421	0.285
Decrese	0.04 (0.04)	-0.12 (0.04)	-0.12 (0.01)	0.698	0.006	>0.8

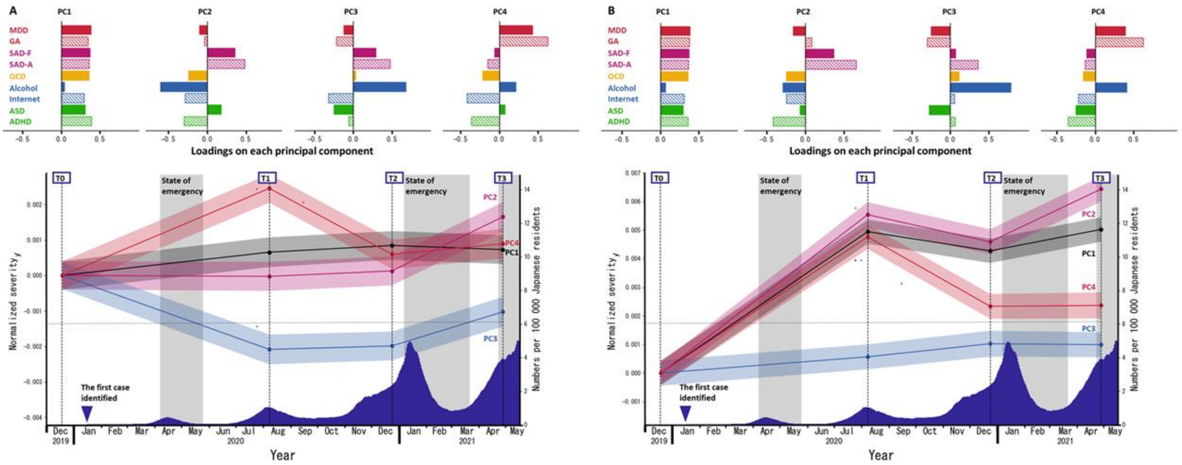

Supplementary Figure 5. A. Each PC loading and trajectory in males **B.** Each PC loading and trajectory in females. Each upper figure shows loadings of psychiatric disorder scores for each principal component. Each lower figure shows trajectories of the average of each PC. The blue area under the curve represents a 15-day moving average of daily new COVID-19 cases per 100 000 Japanese residents. Signs of PCs were arranged so that they followed the main analysis. Asterisks indicate significant changes in PC score from the previous time point (p<.05, Bonferroni-corrected).

MDD: major depressive disorder as measured with the CES-D; GA: general anxiety as measured with the STAI-Y state, SAD-F: fear aspects of social anxiety disorder as measured using the LSAS-F; SAD-A: avoidance aspects of social anxiety disorder as measured with the LSAS-A; OCD: obsessive-compulsive disorder as measured using the OCI; Alcohol: alcohol-related problems as measured using the AUDIT; Internet: internet-related problems as measured with the CIUS; ASD: autism as measured with the AQ; ADHD: attention deficit and hyperactivity disorders as measured using the ASRS

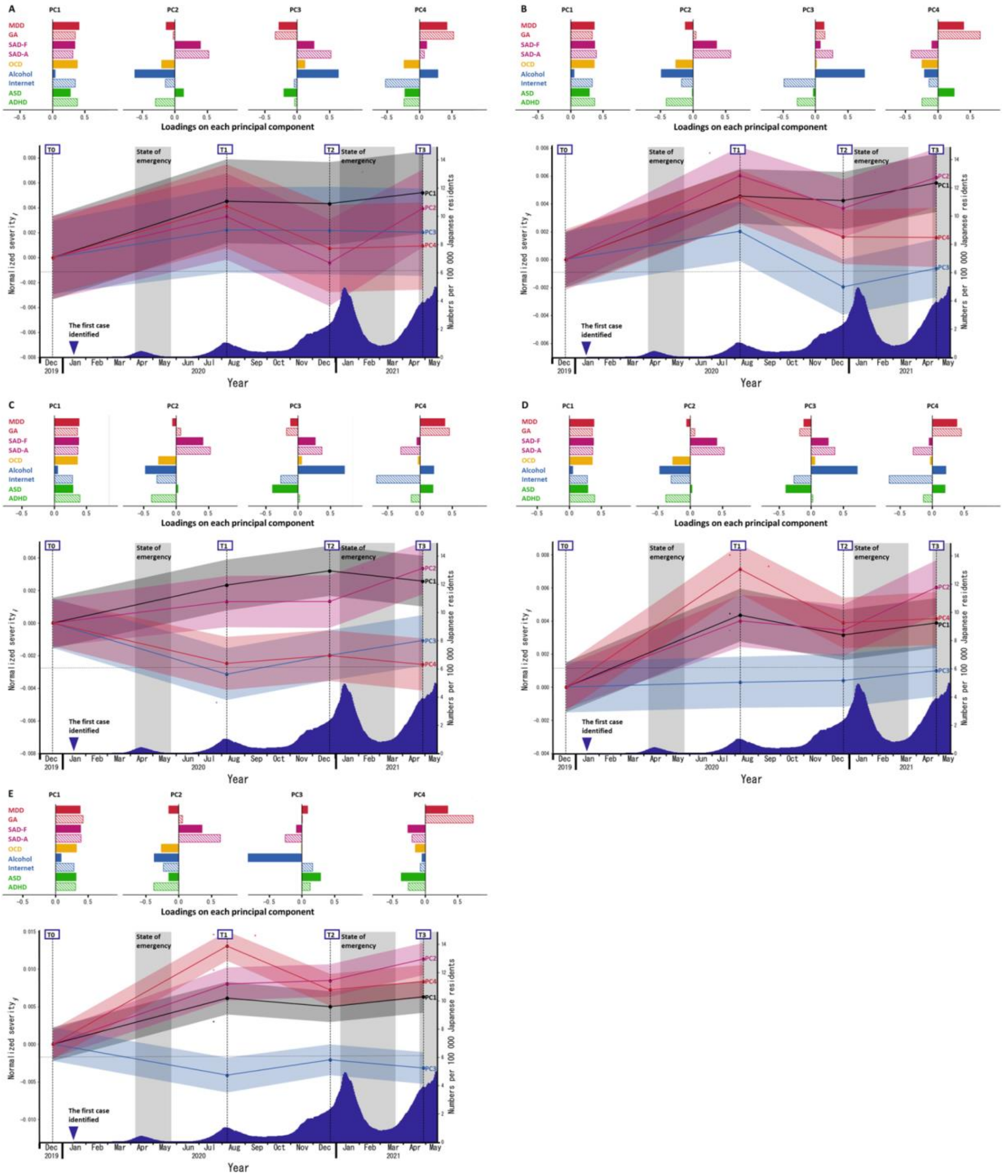

Supplementary Figure 6. A-E. PC loadings and trajectories in each age group (20 ~ 60’s, per 10 years). Each upper figure show loadings of psychiatric disorder scores for each principal component. Each lower figure shows trajectories of the average of each PC. *In figures B and E, the order of PC3 and PC4 are reversed for ease of comparison. The blue area under the curve represents a 15-day moving average of daily new COVID-19 cases per 100 000 Japanese residents. Signs of PCs were arranged so that they follow the main analysis. Asterisks indicate significant changes in PC score from the previous time point (p<.05, Bonferroni-corrected).

MDD: major depressive disorder as measured with the CES-D; GA: general anxiety as measured using the STAI-Y state, SAD-F: fear aspects of social anxiety disorder as measured using the LSAS-F; SAD-A: avoidance aspects of social anxiety disorder as measured with LSAS-A; OCD: obsessive-compulsive disorder as measured with the OCI; Alcohol: alcohol-related problems as measured with the AUDIT; Internet: internet-related problems as measured using the CIUS; ASD: autism as measured using the AQ; ADHD: attention deficit and hyperactivity disorders as measured with the ASRS.

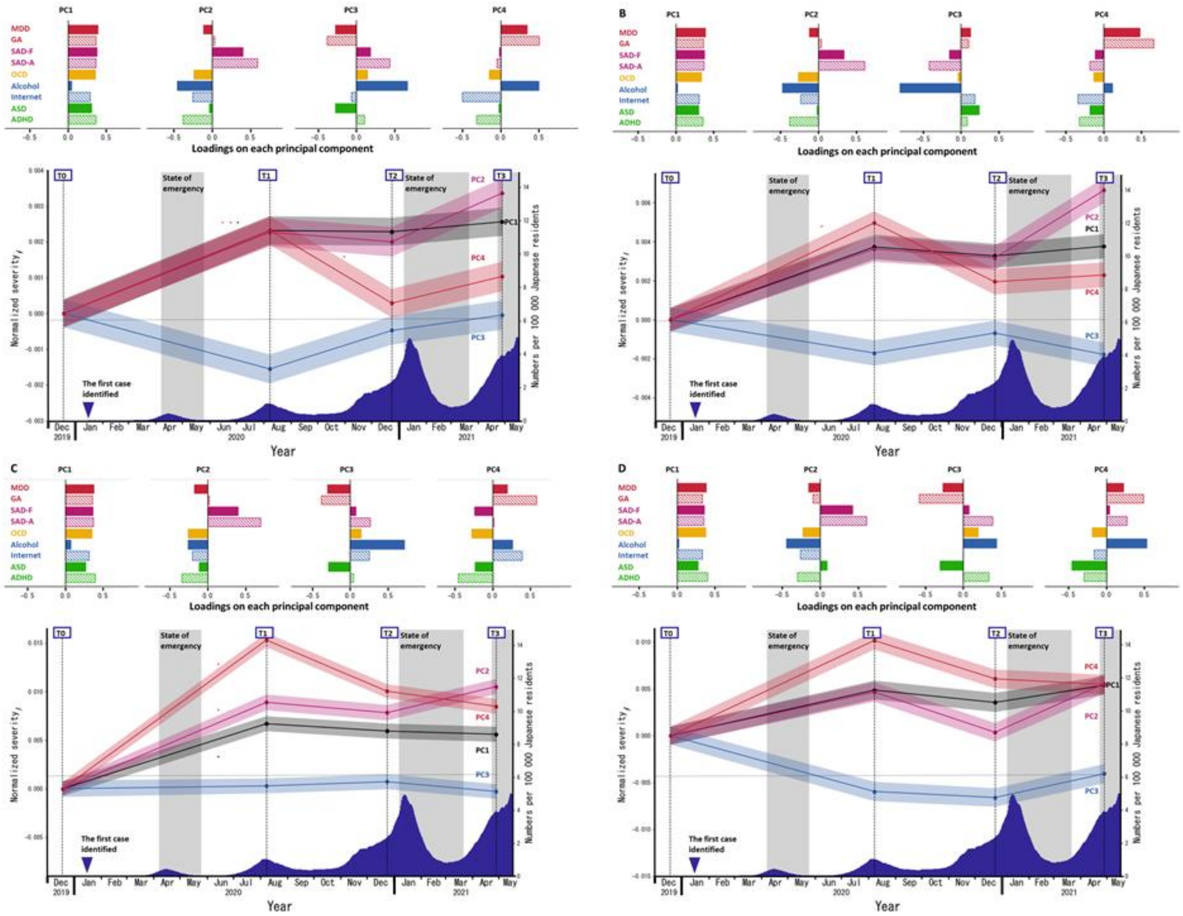

Supplementary Figure 7. PC loadings and trajectories in each quintile group based on the problematic smartphone scores (per 10 A: 0-10, B: 10-20, C: 20-30, D: 30-). Each upper figure shows loadings of psychiatric disorder scores for each principal component. Each lower figure shows trajectories of the average of each PC. *In figures C and D, the order of PC3 and PC4 are reversed for ease of comparison. The blue area under the curve represents a 15-day moving average of daily new COVID-19 cases per 100 000 Japanese residents. Signs of PCs were arranged so that they followed the main analysis. Asterisks indicate significant changes in PC score from the previous time point (p<.05, Bonferroni-corrected).

MDD: major depressive disorder as measured with the CES-D; GA: general anxiety as measured using the STAI-Y state, SAD-F: fear aspects of social anxiety disorder as measured using the LSAS-F; SAD-A: avoidance aspects of social anxiety disorder as measured with the LSAS-A; OCD: obsessive-compulsive disorder as measured with the OCI; Alcohol: alcohol-related problems as measured using the AUDIT; Internet: internet-related problems as measured with the CIUS; ASD: autism as measured using the AQ; ADHD: attention deficit and hyperactivity disorders as measured with the ASRS.

Supplementary References

1 Radloff LS. The CES-D Scale: A Self-Report Depression Scale for Research in the General Population. *Appl Psychol Meas* 1977; **1**: 385–401.

2 SHIMA, S. Reliability and validity of CES-D. *Jpn J Psychiatry Neurol* 1985; **27**: 717–723.

3 Spielberger CD. State-Trait Anxiety Inventory for Adults. 1983. doi:10.1037/t06496-000.

4 Iwata N, Mishima N, Shimizu T, Mizoue T, Fukuhara M, Hidano T *et al.* The Japanese adaptation of the STAI Form Y in Japanese working adults--the presence or absence of anxiety. *Ind Health* 1998; **36**: 8–13.

5 Baker SL, Heinrichs N, Kim H-J, Hofmann SG. The liebowitz social anxiety scale as a self-report instrument: a preliminary psychometric analysis. *Behav Res Ther* 2002; **40**: 701–715.

6 Asakura S, Inoue S, Sasaki F, Sasaki Y, Kitagawa N, Inoue T *et al.* Reliability and validity of the Japanese version of the Liebowitz Social Anxiety Scale. *Seishin Igaku* 2002; **44**: 1077–1084.

7 Foa EB, Kozak MJ, Salkovskis PM, Coles ME, Amir N. The validation of a new obsessive-compulsive disorder scale: The Obsessive-Compulsive Inventory. Psychological Assessment. 1998; **10**: 206–214.

8 Ishikawa R, Kobori O, Shimizu E. Development and validation of the Japanese version of the obsessive-compulsive inventory. *BMC Res Notes* 2014; **7**: 306.

9 Saunders JB, Aasland OG, Babor TF, de La Fuente JR, Grant M. Development of the Alcohol Use Disorders Identification Test (AUDIT): WHO Collaborative Project on Early Detection of Persons with Harmful Alcohol Consumption-II. *Addiction* 1993; **88**: 791–804.

10 Hiro H, Shima S. Availability of the Alcohol Use Disorders Identification Test (AUDIT) for a complete health examination in Japan. *Nihon Arukoru Yakubutsu Igakkai Zasshi* 1996; **31**: 437–450.

11 Meerkerk G-J, Van Den Eijnden RJJM, Vermulst AA, Garretsen HFL. The Compulsive Internet Use Scale (CIUS): some psychometric properties. *Cyberpsychol Behav* 2009; **12**: 1–6.

12 Yong RKF, Inoue A, Kawakami N. The validity and psychometric properties of the Japanese version of the Compulsive Internet Use Scale (CIUS). *BMC Psychiatry* 2017; **17**: 201.

13 Baron-Cohen S, Wheelwright S, Skinner R, Martin J, Clubley E. The autism-spectrum quotient (AQ): evidence from Asperger syndrome/high-functioning autism, males and females, scientists and mathematicians. *J Autism Dev Disord* 2001; **31**: 5–17.

14 Wakabayashi A, Baron-Cohen S, Wheelwright S, Tojo Y. The Autism-Spectrum Quotient (AQ) in Japan: A cross-cultural comparison. *J Autism Dev Disord* 2006; **36**: 263–270.

15 Kessler RC, Adler L, Ames M, Demler O, Faraone S, Hiripi E *et al.* The World Health Organization Adult ADHD Self-Report Scale (ASRS): a short screening scale for use in the general population. *Psychol Med* 2005; **35**: 245–256.

16 Adler LA, Spencer T, Faraone SV, Kessler RC, Howes MJ, Biederman J *et al.* Validity of pilot Adult ADHD Self- Report Scale (ASRS) to Rate Adult ADHD symptoms. *Ann Clin Psychiatry* 2006; **18**: 145–148.

17 Takeda T, Tsuji Y, Kurita H. Psychometric properties of the Japanese version of the Adult Attention-deficit hyperactivity disorder (ADHD) Self-Report Scale (ASRS-J) and its short scale in accordance with DSM-5 diagnostic criteria. *Res Dev Disabil* 2017; **63**: 59–66.

18 Panagiotidi M, Overton P. The relationship between internet addiction, attention deficit hyperactivity symptoms and online activities in adults. *Compr Psychiatry* 2018; **87**: 7–11.

19 Kim K, Ryu E, Chon M-Y, Yeun E-J, Choi S-Y, Seo J-S *et al.* Internet addiction in Korean adolescents and its relation to depression and suicidal ideation: a questionnaire survey. *Int J Nurs Stud* 2006; **43**: 185–192.

20 Hirota T, McElroy E, So R. Network Analysis of Internet Addiction Symptoms Among a Clinical Sample

of Japanese Adolescents with Autism Spectrum Disorder. *J Autism Dev Disord* doi:10.1007/s10803-020-04714-x.

21 Weinstein A, Dorani D, Elhadif R, Bukovza Y, Yarmulnik A, Dannon P. Internet addiction is associated with social anxiety in young adults. *Ann Clin Psychiatry* 2015; **27**: 4–9.

22 Choi S-W, Kim D-J, Choi J-S, Ahn H, Choi E-J, Song W-Y *et al.* Comparison of risk and protective factors associated with smartphone addiction and Internet addiction. *J Behav Addict* 2015; **4**: 308–314.

23 Ha JH, Kim SY, Bae SC, Bae S, Kim H, Sim M *et al.* Depression and Internet addiction in adolescents. *Psychopathology* 2007; **40**: 424–430.
